# Anterior thalamic nucleus local field potentials during focal temporal lobe epileptic seizures

**DOI:** 10.3389/fneur.2024.1419835

**Published:** 2024-06-18

**Authors:** Jaysingh Singh, Jacob A. Miller, Timothy Lucas, Jimmy Yang, Caleb Sollars, Dawn S. Eliashiv, Fabrice Bartolomei

**Affiliations:** ^1^Department of Neurology, The Ohio State University Wexner Medical Center, Columbus, OH, United States; ^2^Department of Biomedical Engineering, The Ohio State University, Columbus, OH, United States; ^3^Depart of Neurosurgery, The Ohio State University Wexner Medical Center, Columbus, OH, United States; ^4^Lead EEG Technician, Neurodiagnostic EEG Lab, The Ohio State University Wexner Medical Center, Columbus, OH, United States; ^5^Department of Neurology, David Gefen School of Medicine, UCLA, Los Angeles, CA, United States; ^6^APHM, Timone Hospital, Epileptology and Cerebral Rhythmology Department, Marseille, France; ^7^Univ Aix Marseille, INSERM, INS, Inst Neurosci Syst, Marseille, France

**Keywords:** deep brain stimulation, local field potential, anterior nucleus of thalamus, temporal lobe epilepsy, DBS

## Abstract

**Objective:**

To analyze the local field potentials (LFPs) in patients with focal drug-resistant epilepsy (DRE) from the anterior nucleus of the thalamus (ANT) during inter-ictal state and seizure state.

**Method:**

ANT stereotactic EEG (SEEG) recordings were studied in four patients with focal temporal lobe epilepsy. SEEG data was classified as inter-ictal and ictal state and sub-categorized into electrographic (ESz), focal aware seizure (FAS), focal with impaired awareness (FIA), or focal to bilateral tonic-clonic seizure (FBTC). LFP was analyzed at 4 Hz, 8 Hz, 16 Hz, 32 Hz, high gamma (100 Hz), and ripples (200 Hz) using spectrogram analysis and a statistical comparison of normalized power spectral density (PSD) averaged during seizures versus pre-ictal baseline segments.

**Result:**

The LFP recordings were analyzed for 162 seizures (127 ESz, 23 FAS, 6 FIA, and 6 FBTC). Based on time-frequency data (spectrogram), a broad band of activity, occurring between 2 and 6 Hz and centered at 4 Hz, and thin-band activity occurring specifically at 8 Hz on the frequency spectrogram were observed during the inter-ictal state. Statistically significant changes in LFP-PSD were seen for FAS, FIA, and FBTC. We observed a significant gain in LFP at the lower frequency band during FAS at 4 Hz, FIA, and FBTC at 4, 8, and 16 Hz while also observing increases at higher frequencies during FBTC at 100 and 200 Hz and a decrease during FAS seizures at 32 Hz. In contrast, no significant change in LFP power was seen for electrographic seizures.

**Interpretation:**

Our observations from a limited dataset indicate that all clinical seizure types, but not electrographic seizures, caused a change in ANT-LFP based on the magnitude of the associated power spectral density (PSD). Future work will be needed to validate the use of ANT-LFP at these frequencies as accurate measurements of seizure occurrence and severity. This work represents a first step toward understanding ANT thalamic LFP patterns during focal seizures and developing adaptive DBS strategies.

## Highlights

- There is limited data characterizing thalamic local field potential (LFP) patterns during focal epileptic seizures.
- Here we analyzed LFP from the anterior nucleus of the thalamus (ANT) and observed consistent broad activity at 4 Hz and thin-band activity at 8 Hz during resting or inter-ictal state.
- We observed a significant gain in LPF at the lower frequency band during FAS (4 Hz), FIA (4, 8, and 16 Hz), and FBTC (4, 8, and 16 Hz) while also observing increases at a higher frequency during FBTC seizures (100 and 200 Hz). In contrast, no significant change in LFP power was seen for electrographic seizures.
- Further investigations are needed to evaluate the clinical use of LFP in ANT-DBS programming for the management of seizures.

## Introduction

Deep brain stimulation (DBS) is a neuromodulation technique that has shown positive results in patients with drug-resistant epilepsy (DRE), which accounts for 30% of all cases of epilepsy (1). For adults with focal DRE, the DBS targeting the anterior nucleus of the thalamus (ANT) has shown promising findings with 69% median seizure frequency reduction and 68% responder rate, defined as ≥50% reduction in seizure frequency, at five years (2). Several potential DBS targets, like the centromedian nucleus and hippocampus, have shown mean seizure reduction of 73.4 and 67.8%, respectively (3). In contrast, the benefit of stimulation of the cerebellum (4), sub-thalamic nucleus (5), caudate nucleus (6), posterior hypothalamus (7), hippocampal fornix (8), and nucleus accumbens (9) remains uncertain.

A new generation of DBS devices provides the unique opportunity to record signals directly from the thalamic nucleus, thus supporting the study of thalamic neurophysiological signatures during focal epileptic seizures and potentially guiding DBS therapy. ANT-DBS macro electrodes can record the pre-and postsynaptic activity of neural populations surrounding the lead, known as local field potentials (LFPs). The LFP frequency bands were defined in a range of delta (0–3 Hz), theta (4–7 Hz), alpha (8–12 Hz), beta (13–35 Hz), gamma (31–200 Hz), and high-frequency band (>200 Hz). Following the extensive experience gained with DBS in Parkinson’s disease, multiple groups have shown that specific LFP oscillatory rhythm in the beta band (13–30 Hz) was correlated well with the patient’s symptoms (especially akinetic and rigid symptoms of PD) (10, 11). This has inspired a concept of adaptive DBS, which uses neurophysiological signals as feedback to guide DBS programming in Parkinson’s disease (12). However, ANT-DBS neuromodulation in patients with focal DRE remains an open-loop system, and programming is based on stimulation parameters from the pivotal SANTE trial. There is currently a lack of LFP data in patients with focal DRE.

This work aims to analyze the LFP signals in patients with drug-resistant focal temporal lobe epilepsy from ANT stereo EEG intracranial recording during inter-ictal and different types of seizures (electrographic vs. clinical).

## Methods

### Patient information

We reviewed patients who underwent stereo-EEG (SEEG) exploration from January 2019 to March 2023 for epilepsy surgery evaluation and had at least one intracranial electrode implanted in the anterior nucleus of the thalamus. The Institutional Review Board approved this study at the Ohio State University Wexner Medical Center. Clinical data and SEEG recordings, including recorded focal epileptic seizures, were collected and analyzed (Table 1).

**Table 1:** Summary of patient characteristics and seizure types.

Patient No.	Age	Sex	Epilepsy type	Etiology	Epilepsy duration (years)	ESz	FAS	FIA	FBTC	Total No. of seizures
1.	24	M	Left mesial -TLE	MTS	23	16	0	2	1	19
2.	63	F	Right mesial-TLE	MTS	15	112	6	0	0	118
3.	32	M	Left mesio-neocortical TLE	TBI	5	1	0	1	3	5
4.	35	M	Right temporo-insular onset epilepsy	Unknown	28	0	17	3	2	22

TLE, temporal lobe epilepsy; MTS, mesial temporal lobe sclerosis; TBI, traumatic brain injury; ESz, electrographic seizure; FAS, focal aware seizure; FIA, focal seizure with impaired awareness; FBTC, focal to bilateral tonic-clonic seizure.

SEEG recording was performed using DIXI intracerebral multiple contact electrodes (8–18 contacts, length 2 mm, diameter 0.8 mm, 1.5 mm apart), and SEEG signals were recorded on a Natus system sampled at 1,024–2,048 Hz with 16-bit resolution. The decision to include ANT-thalamic depth electrodes as a part of SEEG monitoring was determined based on multidisciplinary epilepsy surgery team recommendations, which were based on features such as poorly lateralized seizure onset or the presence of bilateral abnormalities on surface EEG and/or discordant imaging abnormalities, suggesting that patients’ treatment could lead to thalamic neuromodulation (13). Electrodes were placed intracerebrally according to the Talairach stereotactic method, and frontal trans ventricular trajectory was adopted for ANT-SEEG electrode placement. ANT-thalamic implantation was unilateral in all patients. The anatomical targeting of ANT-SEEG depth electrodes was re-confirmed using CURRY 9 software, whereby post-SEEG CT head images were fused on Pre-Op MRI brain images, and ANT contact locations were established.

SEEG raw data were analyzed and classified as inter-ictal state or resting state (absence of seizures) and seizure state, which was sub-categorized into electrographic seizure (ESz), focal aware seizure (FAS), focal with impaired awareness (FIA), and focal to bilateral tonic-clonic (FBTC) seizure types. We defined electrographic seizures as epileptiform discharges >2.5 Hz for at least 10 s or any pattern with definite evolution and for at least 10 s with the absence of objective or subjective clinical manifestations. SEEG seizure data were marked with the electrographic onset of a seizure and the start of ANT electrode involvement (labeled as ANT). In cases of FBTC seizure, we also annotated the clinical onset of generalized tonic-clonic (GTC) onset to avoid excessive external noise artifact in LFP signal analysis. All the annotations were marked by CS and reviewed by JS. Once the SEEG data was reviewed and annotated, only SEEG—ANT electrode data was converted into EDF (European data format) files, down-sampled to 512 Hz, and transferred to MATLAB (The MathWorks, Inc., United States) for LFP signal analysis. The sample size is presented as the amount of seizures/number of patients.

### Signal processing and artifact removal

Continuous time-domain recordings, recorded continuously for the entire stay of the patient, were segmented from the bipolar configuration of the deepest ANT electrodes (ANT1–ANT2) based on periods of manual signal pruning by a trained technologist (CS). Periods without signal were removed and each recording was separated into files of continuously recorded data. A notch filter was then applied to the time domain signal at 60, 120, and 180 Hz to remove power line interference and co-frequencies. The signal was high passed at 0.5 Hz to remove low-frequency artifacts, and a cutoff threshold of 1,500 μV was applied (12). Afterward, notable remaining artifacts, most commonly by cable failure/disconnection and high electrode impedance, were removed manually.

### Spectrogram analysis

Patient time domain data was segmented into groups based on SEEG recording annotations. For each seizure, a section of the original SEEG recording with the length of the seizure ± 60 s was isolated from the recording of the entire patient stay. These sections of SEEG were subsequently illustrated in a time-frequency map by processing each segment individually using the MATLAB *spectrogram* function, calculating the power spectral density (PSD) via a short-time Fourier transform with an applied Hanning window with 1 s length, 0.25 s overlap, and using 512 points (i.e., the window length and sampling frequency). This provides a measurement of frequency-normalized PSD (dB/Hz) with a frequency resolution of 1 Hz. Spectrograms were reviewed to identify “frequencies of interest,” defined as the active frequencies during ictal vs. inter-ictal periods, which were then used as the starting point for analysis of frequency-specific activity.

### Isolating frequency-specific activity

To provide a comparison of LFP across patients for ictal vs. inter-ictal periods, the pruned SEEG signal was zero-padded and then the data was normalized by calculating the PSD once for each patient over their entire multi-day stay (patients 1–3: 7 days, patient 4: 5 days) using the MATLAB *spectrogram* function to take the short-time Fourier transform as done prior. This resulted in a time-dependent measure of PSD for each frequency within the sampling range (up to 256 Hz) with a 1 Hz resolution. The PSD was then averaged over a range +/−2 Hz centered at each frequency of interest (i.e., 4 Hz +/−2 Hz, 8 Hz +/−2 Hz, etc.) providing a time-dependent PSD with a frequency resolution similar to what a physician would observe via a DBS recording of ANT local field potentials in-clinic. Because PSD was compared between patients with potential differences in ANT electrode impedance, altering the recorded amplitude of the signal, a *z*-score normalization was applied to the PSD for each frequency of interest, individually for each patient. This resulted in a normalized distribution of PSD for each frequency, centered at 0 and with a standard deviation of 1. Normalizing in this way allowed a direct comparison of PSD magnitude across patients, providing a clear point of comparison (the median value, zero) for PSD magnitude between different types of seizures.

**Figure 1:**
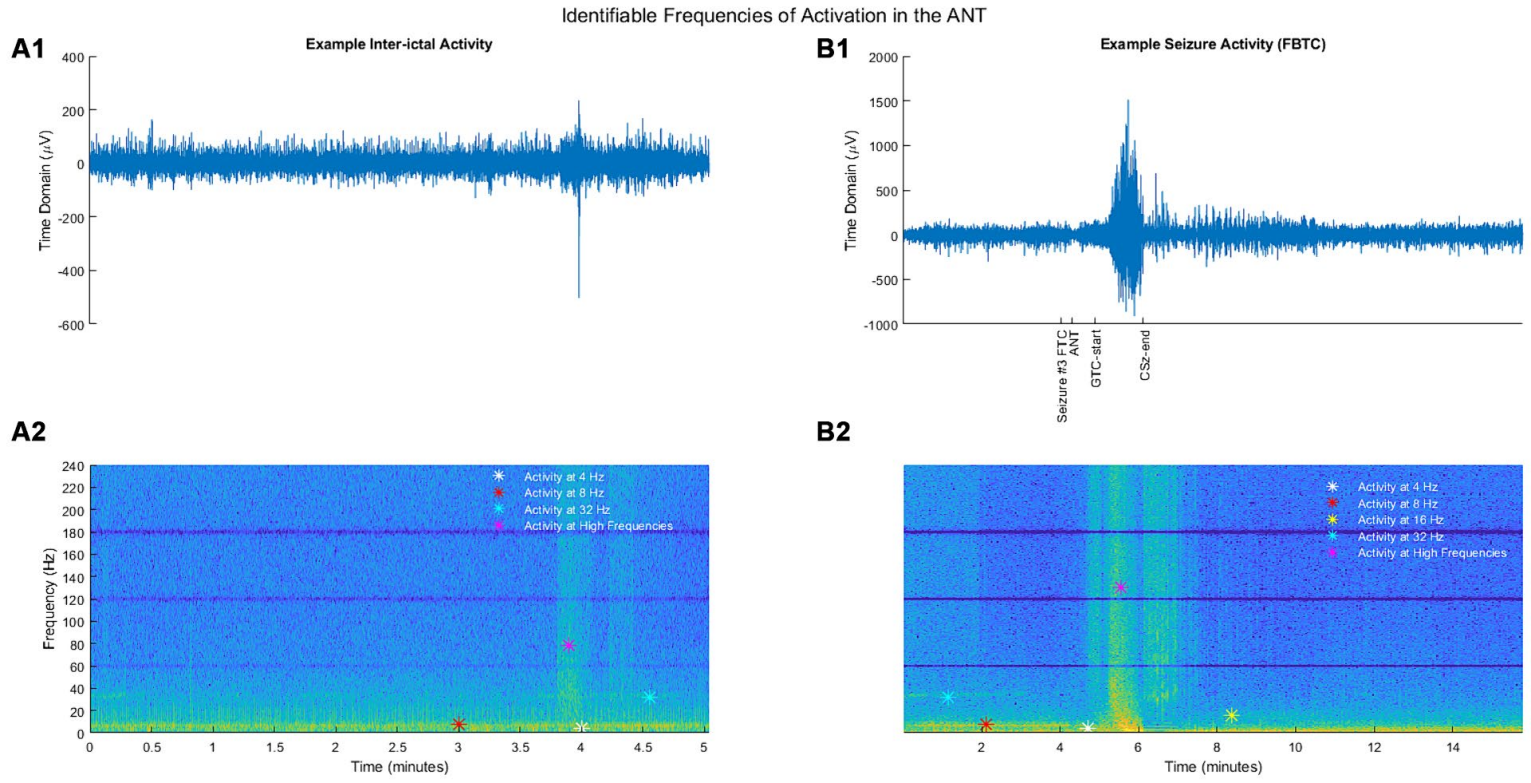
**(A1)** Example segment of inter-ictal activity in the time domain. **(B1)** Example segment of seizure activity (FBTC) in the time domain. **(A2)** Inter-ictal activity from **(A1)** illustrated as a spectrogram. **(B2)** Seizure activity from **(B1)** illustrated as a spectrogram. For spectrograms, the *y*-axis represents the frequency (Hz), and the color depicts a frequency-normalized signal (unitless) for a given time and frequency.

We compared PSD at a subset of frequencies of interest based on previous publications or findings from the spectrogram analysis. Four hertz and Eight hertz were chosen to encompass frequencies of interest based on initial observation by Fasano et al. (14). Other frequencies in beta (16 Hz), gamma (32 Hz), high gamma (100 Hz), and ripples (200 Hz) range were chosen based on the spectrogram analysis (example shown in Figure 1).

To establish a baseline for statistical comparison, seizure data was compared to a 40 s segment ending 2 min prior to seizure onset, similar to that used by Grinenko et al. (15). Although research has found that changes in intracranial EEG activity can occur minutes before seizure onset, this is thought to be highly non-linear and does not necessitate changes in LFP magnitude at particular frequencies (16). Further, a broad-band investigation of changes in the ANT from pre-seizure to post-termination in patients with mesial temporal lobe seizures found ANT-LFP does not change until the seizure onset period, defined as the first 10 s after ictal onset (equivalent to the seizure start in this study) based on the PSD (17). To provide additional support for the use of these pre-ictal segments as a baseline, we analyzed the distribution of average normalized PSD at each frequency of interest during pre-ictal segments (Supplementary Figure S1). We find the pre-seizure LFP segments were centered around 0, regardless of the frequency, with the vast majority of values being within 1 standard deviation of the, mean, providing additional evidence for normal ANT activity during these pre-ictal periods.

**Figure 2:**
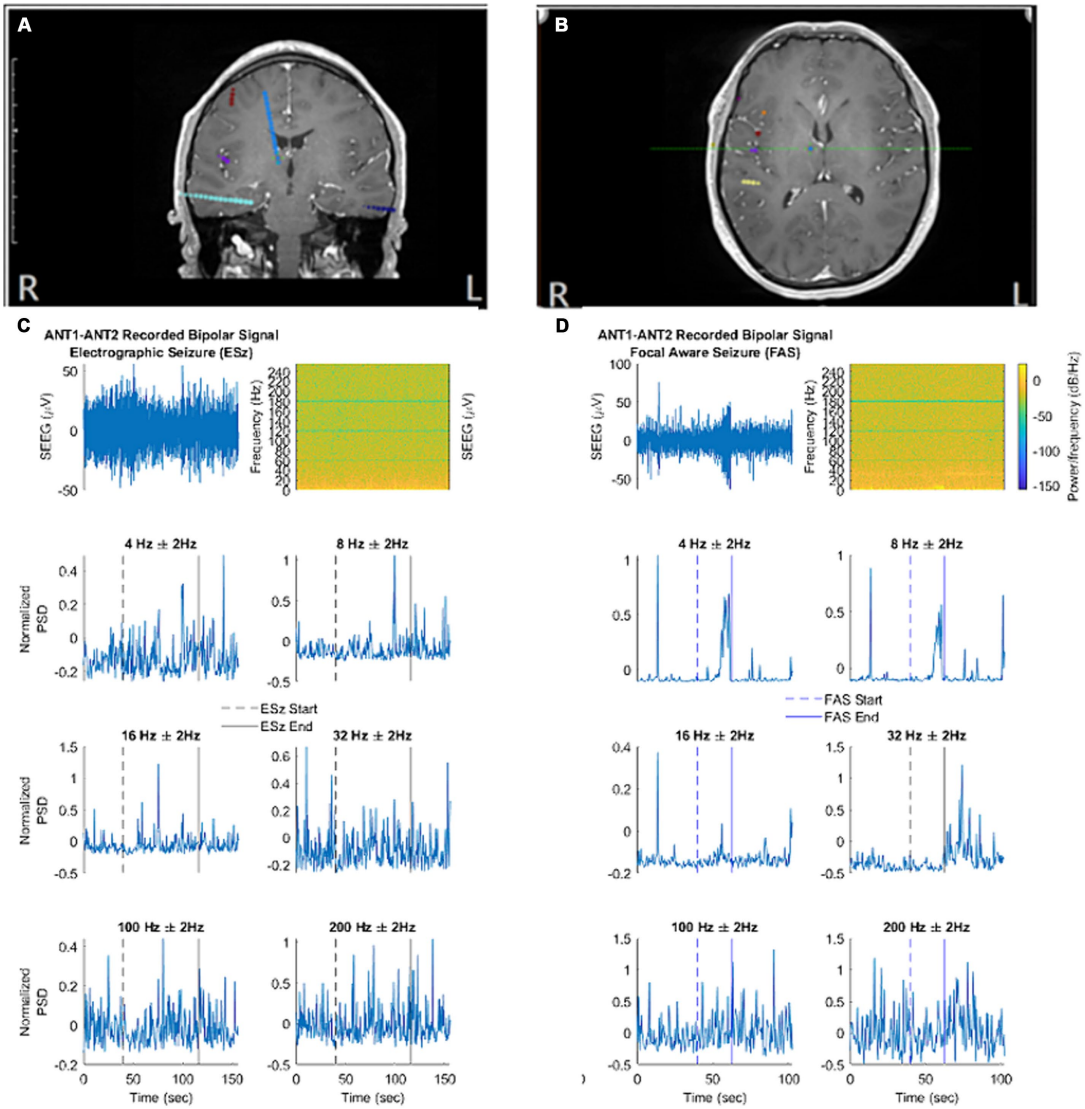
**(A,B)** Localization of ANT contacts on co-registered MRI brain images (blue contacts). **(C,D)** Illustrate the filtered signal in the time domain, spectral analysis, and normalized spectral power at each frequency of interest. **(C)** An example electrographic seizure (ESz) taken from patient 1. **(D)** An example focal aware seizure (FAS) taken from patient 4. **(A)** - correspond to first MRI image. **(B)** - correspond to second MRI brain image. **(C, D)** correspond to EEG/Spectrogram.

**Figure 3:**
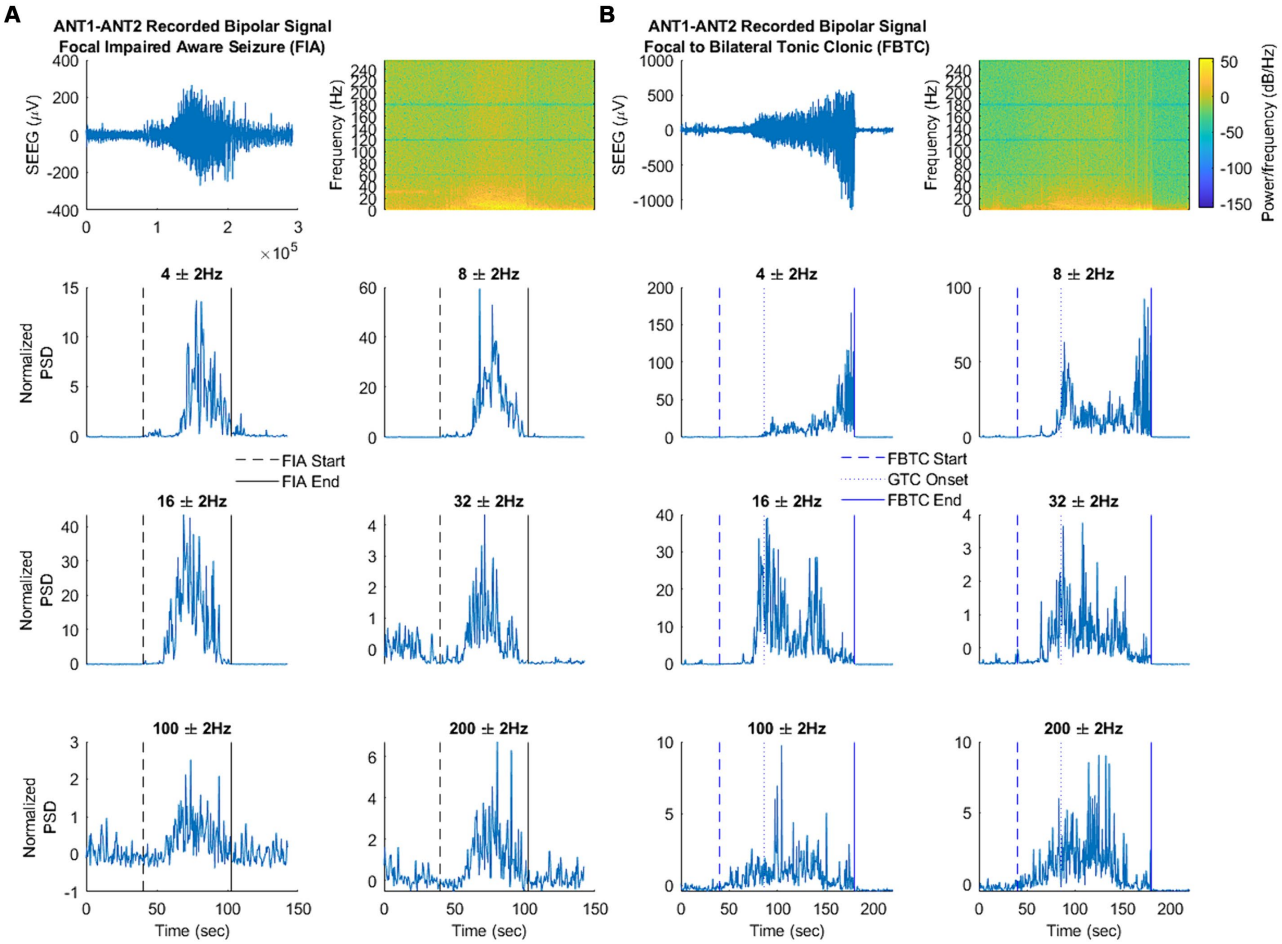
**(A,B)** Illustrate the filtered signal in the time domain, spectral analysis, and signal power at each frequency of interest. **(A)** An example focal seizure with impaired awareness (FIA) taken from patient 1. **(B)** An example focal to bilateral tonic–clonic seizure (FBTC) taken from patient 1. Note that changes in LFP were only analyzed from FBTC start to GTC onset.

Because the method of statistical comparison requires a baseline segment beginning 160 s before seizure onset, a seizure was not included in the analysis if the obtainment of this “baseline segment” was unavailable due to signal pruning. An example of the filtered SEEG signal taken from the ANT, as well as the calculated frequency-specific PSD at each frequency of interest is illustrated for ESz and FAS (Figures 2C,D, respectively), and FIA and FBTC (Figures 3A,B, respectively).

To determine significant changes in frequency-specific LFP during ictal periods, the normalized PSD was averaged over the 40 s pre-ictal window for the baseline segment, as well as from seizure start to end for the ictal activity. The exception to this process was during FBTC, which was only averaged from seizure start to GTC-onset to avoid movement artifact. The average normalized PSD was then compared between baseline and ictal segments, subcategorized by seizure type (ESz, FIA, FAS, and FBTC), for each frequency of interest. This process was initially done across all four patients and then repeated for each patient individually. Testing for significant differences between groups was calculated via a Kruskal–Wallis test, considering *p* < 0.01 as statistically significant. This was followed by Dunnett’s test as appropriate to determine which seizure type was significantly different from the baseline LFP.

## Results

Four patients were identified who had ANT-SEEG exploration during the phase II surgical evaluation and a total of 162 seizures met the study criteria and were included in the final analysis. A summary of patient characteristics and seizure types (ESz, FAS, FIA, and FBTC) is shown in Table 1.

### Spectrogram analysis: ANT active frequencies

Based on spectrogram review, the ANT frequency during the inter-ictal state was marked by consistent broad activity at 4 Hz (theta), thin-band activity at 8 Hz (alpha), inconsistent thin-band activity at 32 Hz (gamma), and sporadic activity in the broader ranges of high gamma and more broadly of ripples (Figure 1A2). No identifiable consistent change in spectral power occurred during ESz (*n* = 127/3). During FIA seizures (*n* = 6/3), there was evidence of a consistent increase in activity at 4 and 8 Hz, as well as an inconsistent increase in high gamma and ripples. FBTC seizures (*n* = 6/3) had a consistent increase in PSD in the range of high gamma and ripples. We also noted an alteration via discontinued activity at 32 Hz before some FAS (n = 23/2), FIA, and FBTC seizures began (Figures 1B2, 3A).

### Statistical analysis

**Figure 4:**
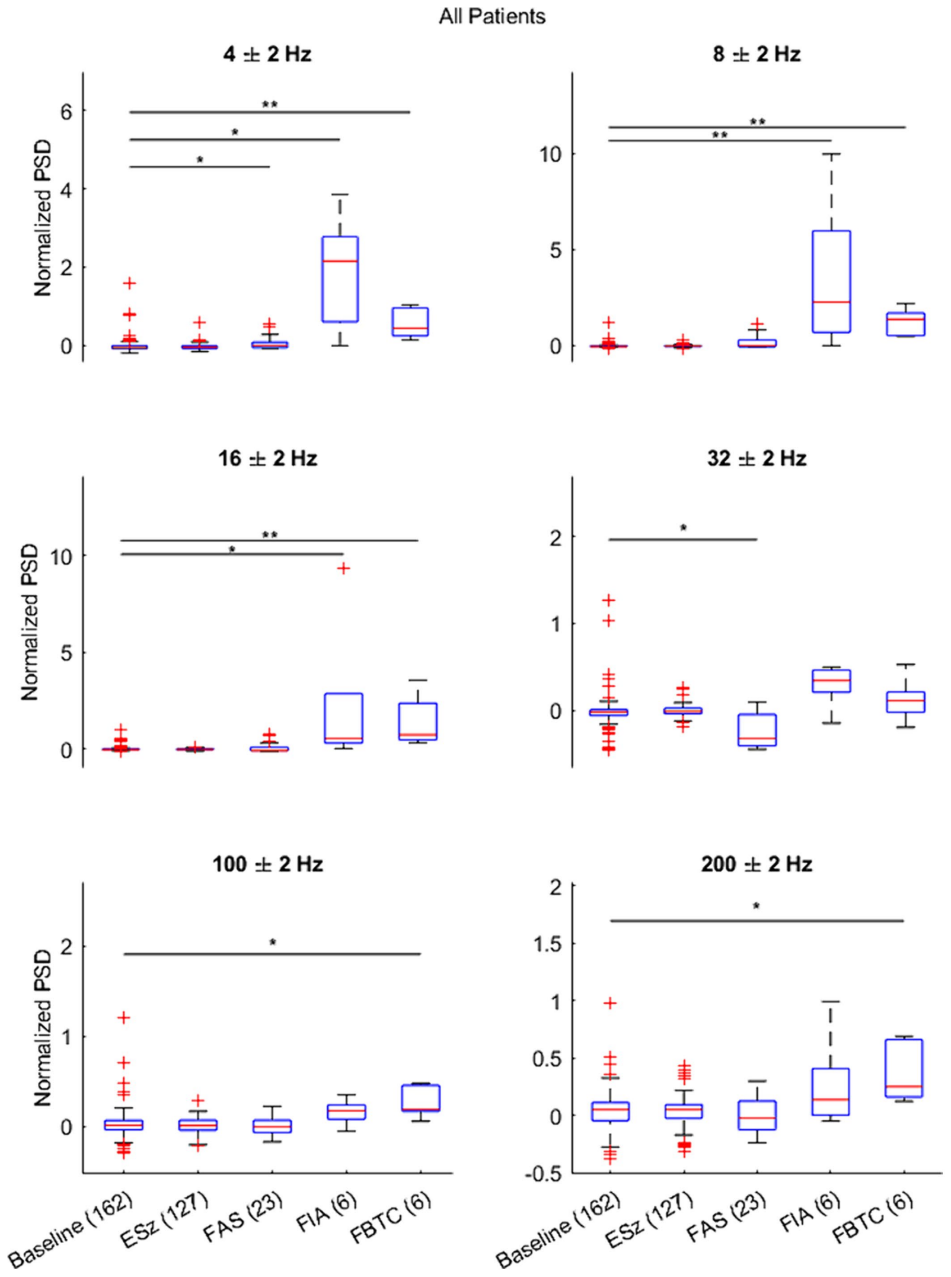
Results of Dunnett’s test comparing the distribution of the average normalized power spectral density (PSD) during each seizure type (ESz, FAS, FIA, and FBTC) versus the matched baseline segment taken 2 min pre-ictal (PreSz). Results indicate changes in ANT-LFP during FAS, FIA, and FBTC seizure types, but not ESz. ^*^p < 0.01 and ^**^p < 0.001.

Results of the Kruskal–Wallis test and subsequently Dunnett’s test to evaluate differences between the average normalized ictal and baseline segments are illustrated across all patients in Figure 4. Changes in LFP during ictal periods depended on the clinical seizure categorization, supporting the unique trends seen while analyzing ictal spectrograms. Notably, no differences were seen between ESz and baseline, supporting the idea that the ANT does not become involved in purely electrographic seizures. ANT-LFP increased at 4 Hz during all clinical seizures, although the largest increase occurred during FIA seizures. Increases in LFP occurred at 8 and 16 Hz during FIA and FBTC seizures, but not FAS. Although all clinical seizures appeared to have, at least in some cases, decreased or discontinued activity at 32 Hz based on spectrogram analysis, this change was only significant during FAS. We observe that an increase in high gamma and ripples activity occurs uniquely during FBTC seizures.

When repeating the statistical test for each patient individually (Supplementary Figures S2–S5), we found no significant differences between seizure and baseline LFP. This can likely be attributed to the small sample sizes for clinical seizures (FIA, FAS, and FBTC) in individual patients, provided these were the only seizure types to change significantly when comparing LFP activity from all four patients together.

**Figure 5:**
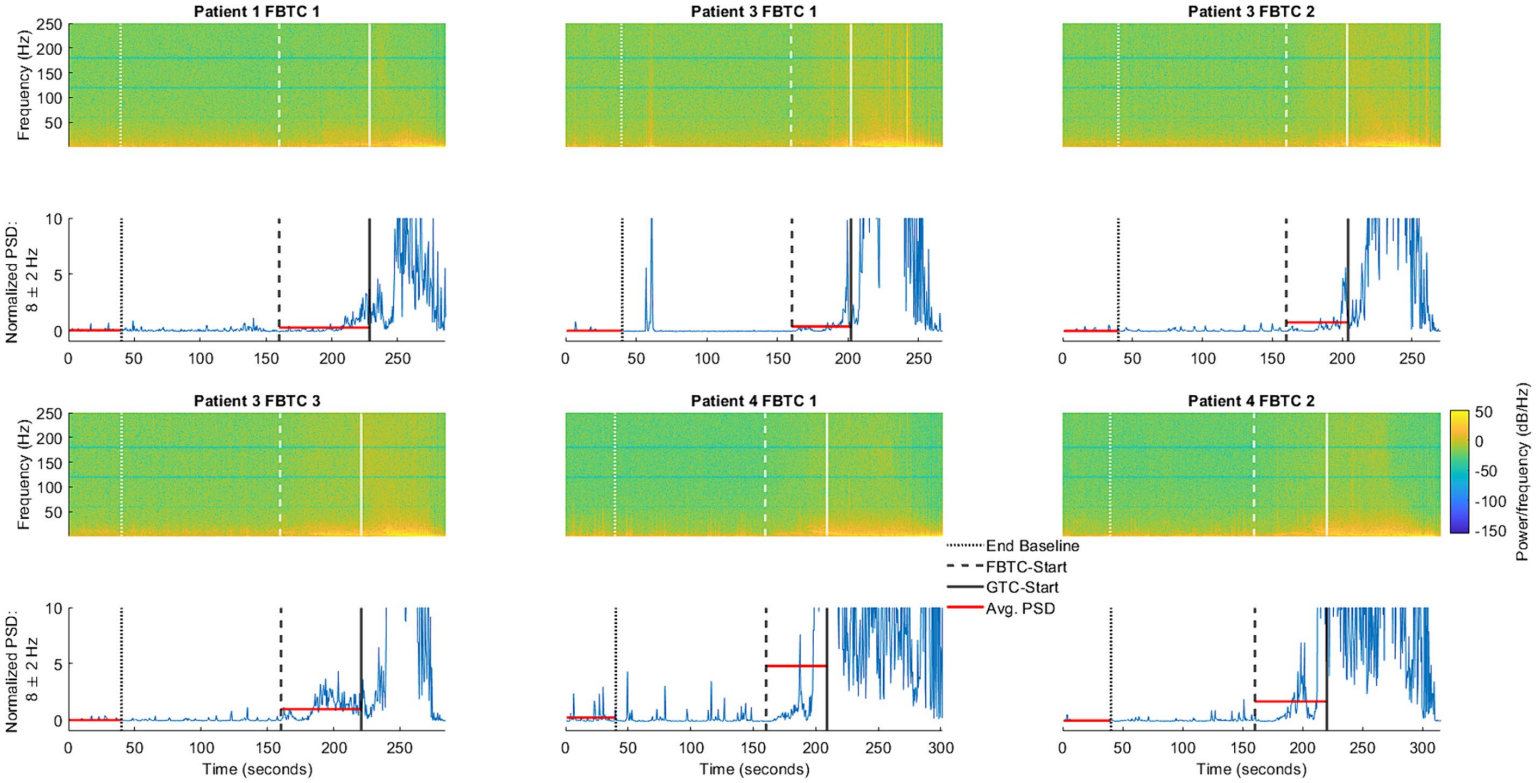
Illustration of ANT activity during the baseline period, occurring in the 40 s ending 2 min before FBTC-start, compared to the six FBTC seizures included in the study. Also shown is the normalized power spectral density (PSD, shown in blue) at 8 Hz—one of the frequencies found to be significantly elevated during FBTC seizures. The horizontal lines (shown in red) represent the average PSD during the baseline and seizure segments, as used in the statistical analysis shown (Figure 4).

To support findings based on the average PSD, we provide a comparison of the time-frequency activity during FBTC seizures with the normalized PSD and its average at 8 Hz during the ictal and corresponding baseline periods (Figure 5), a frequency found to be significantly elevated during FBTC seizures (Figure 4). Based on the time-frequency analysis, the increase in average PSD at 8 Hz is part of elevated activity occurring more broadly between the upper delta, alpha, and lower theta regions, which has a temporary peak just before tonicity begins. This aligns with results from our statistical analysis illustrating the PSD significantly increases at 4, 8, and 16 Hz during FBTC seizures relative to baseline (Figure 4).

## Discussion

The additional sampling of the ANT in the cohort of DRE patients implanted with depth electrodes as part of their clinical evaluation offered a unique opportunity to study the patterns and involvement of ANT in the epileptogenic network. Our work to our knowledge is one of the first few studies systematically characterizing the ANT thalamic local field potential during inter-ictal and ictal states of focal seizures. We found specific oscillatory rhythms across all clinical seizure types, such as enhanced synchrony in the lower theta (4 Hz) during FAS, whereas alpha (8 Hz) and beta (16 Hz) band during the focal seizure with impaired awareness and focal to bilateral tonic-clonic seizures. In contrast, no significant change in LFP power was seen for electrographic.

ANT thalamic theta waves are commonly seen during certain stages of sleep, primarily triggered in the anteroventral subnucleus of ANT by the hippocampal input (18, 19), and this rhythm has been implicated in the mechanism of synaptic plasticity in the hippocampal formation and retrosplenial cortex. During the awake state, the prevalence of theta rhythm is affected by neurological disorders such as Parkinson’s disease (19, 20) and pain (21). The relationship of thalamic theta waves to epileptic seizures is rather intriguing. Genetic models of absence seizures show that spike-wave discharges evolve from medium voltage 5–9 Hz oscillations in the thalamocortical system (14) and that this abnormal activity depends on the expression of T-type channels in thalamocortical cells (22). Such oscillations are not sufficient to initiate spike-and-wave discharges but may participate significantly in regulating the recurrence of the spike-and-wave complex. Studies by Tyvaert and Gotman et al. (23) with simultaneous EEG recordings and functional MRI in patients with idiopathic generalized epilepsy show that both the ANT and the centromedian/parafascicular (Cm/Pf) nucleus are activated during generalized spike-and-wave discharges. The activity of the corticoreticular Cm/Pf preceded that of the ANT, suggesting that the Cm/Pf may be involved in epileptic discharge initiation or early propagation, while the ANT may have a role in its maintenance (24). We also observed significant LFP power gain at higher frequencies (high gamma and ripples) during FBTC seizure. Pizzo et al. (20) also had similar observation in some cases where seizure onset pattern showed high frequency discharges in pulvinar nucleus of thalamus, reaching a maximum of 190 Hz. This raises the possibility that focal epileptic seizures may arise from an abnormal cross-frequency modulation, in which enhanced synchrony of thalamic theta oscillations could influence the generation of pathological beta or gamma activity (21).

ANT-DBS features brain-sensing technology that can record LFP. Programming of the devices requires choosing a frequency of interest to be monitored over time (14). Considering the feasibility of DBS to record chronic LFP, our findings could support clinicians choosing the LFP frequency peaks. Chronic LFP monitoring may allow us to study the circadian and ultradian patterns outside the clinic (22) and potentially quantify the seizure burden in a real world context.

There is growing evidence supporting the neuromodulation of anterior (ANT) and centromedian (CM) thalamic nuclei using responsive neurostimulation (RNS) for the management of intractable epilepsy, with reported ≥50% seizure control in ~65% of patients (2). The effectiveness of RNS can be explained by chronic stimulation-induced modulation of the epileptic network rather than by direct effects on each detected seizure (24). Our ANT-LFP findings could aid in developing chronic RNS neuro-modulation strategies targeting ANT.

Nevertheless, our study has notable limitations, including the limited number of patients with focal epilepsy and extra-temporal lobe epilepsy. In the limited cohort, we recorded six FIA and FBTC seizures, with an equivalent number of associated baseline segments. Due to the limited sample sizes for these seizures and the associated pre-seizure segments, we combined baseline segments from all seizure types during the statistical analysis, potentially introducing bias if the baseline segments behave differently for each seizure type. Future work with a larger patient cohort will investigate differences in the pre-seizure baseline period based on which seizure type is bing preceded. Another limitation was that our patients in this cohort had ANT sampled on the side that was ipsilateral to the main hypothesis as determined by our consensus conference. Contralateral ANT was not included in our cohort. Another limitation is that not all thalamic subnuclei possibly involved in the temporal lobe seizures have been explored in our study, e.g., the Pulvinar nucleus, which is found to be connected to the temporal lobe and plays a role in the maintenance and propagation of focal temporal lobe seizure (20, 25). The centromedian nucleus which may play a role in the propagation and termination of temporal lobe seizures was not sampled (26). As we were limited by the sampling rate of our macroelectrodes, very high frequency signals could not be characterized. Unlike in Parkinson’s disease, where peak LFP activity corresponds to the patient’s clinical symptoms, LFP activity measured in our study does not reflect the different phases of seizure evolution. Our findings should be considered preliminary observations and must be confirmed by future prospective studies.

## Data availability statement

The raw data supporting the conclusions of this article will be made available by the authors, without undue reservation.

## Ethics statement

The studies involving humans were approved by the Ohio State University Institutional Review Board. The studies were conducted in accordance with the local legislation and institutional requirements. Written informed consent for participation was not required from the participants or the participants’ legal guardians/next of kin because this was retrospective analysis of existing data.

## Author contributions

JS: Conceptualization, Methodology, Writing – original draft, Writing – review & editing. JM: Writing – original draft, Writing – review & editing. TL: Writing – review & editing. JY: Writing – review & editing. CS: Writing – review & editing. DE: Writing – review & editing. FB: Writing – review & editing.
