# Supplementary material for: Anterior thalamic nucleus local field potentials during focal temporal lobe epileptic seizures
Source: Front Neurol. 2024 Jun 18;15:1419835. doi: 10.3389/fneur.2024.1419835 (PMC11221306; doi:10.3389/fneur.2024.1419835)
Supplement: Supplementary file 1 [file Image_1.pdf]

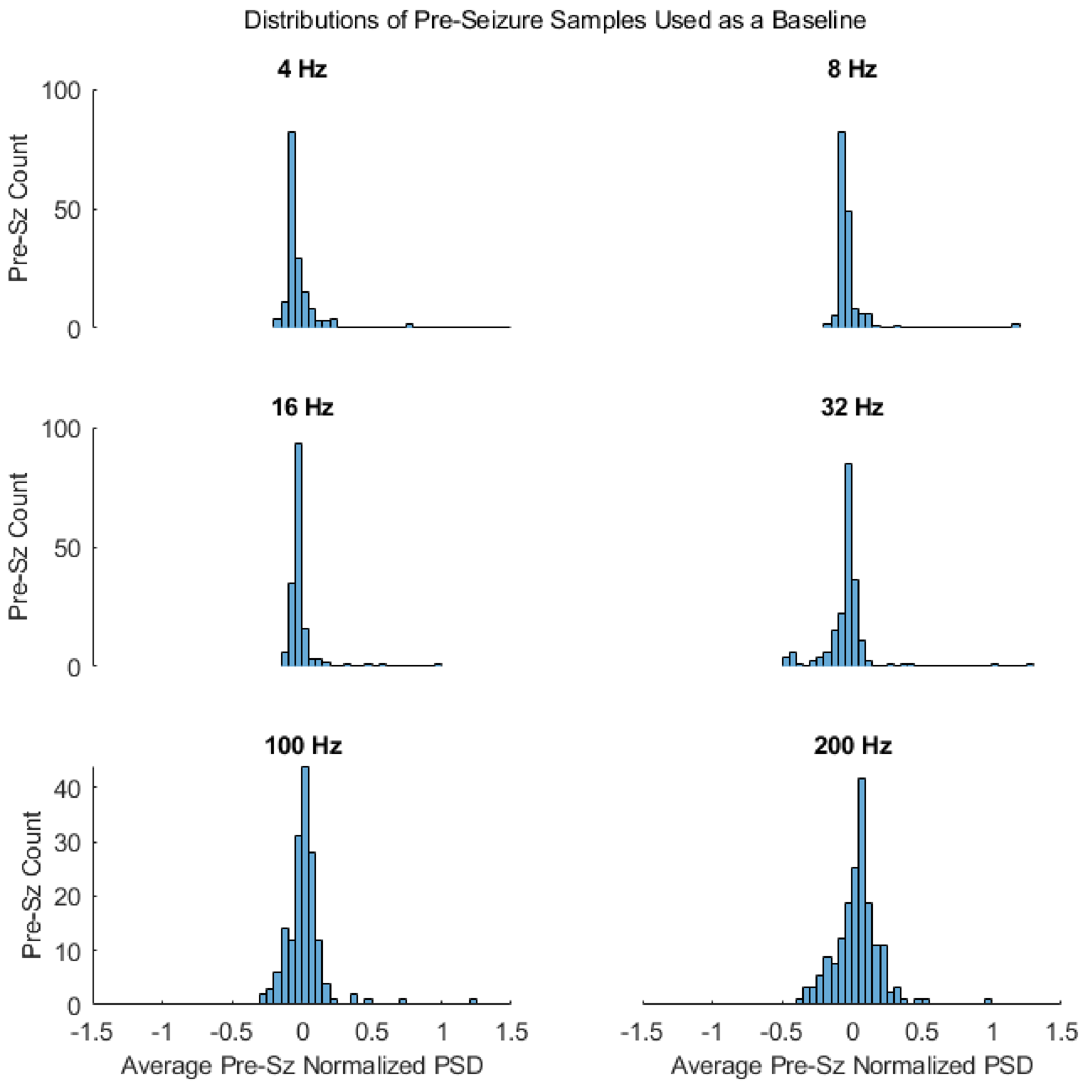

Figure S1: Distribution of average normalized pre-seizure PSD used as a baseline for comparison. All distributions are centered at 0 with a normal distribution, albeit with differing magnitudes of variance. Notably, the number of outliers in each distribution was limited.

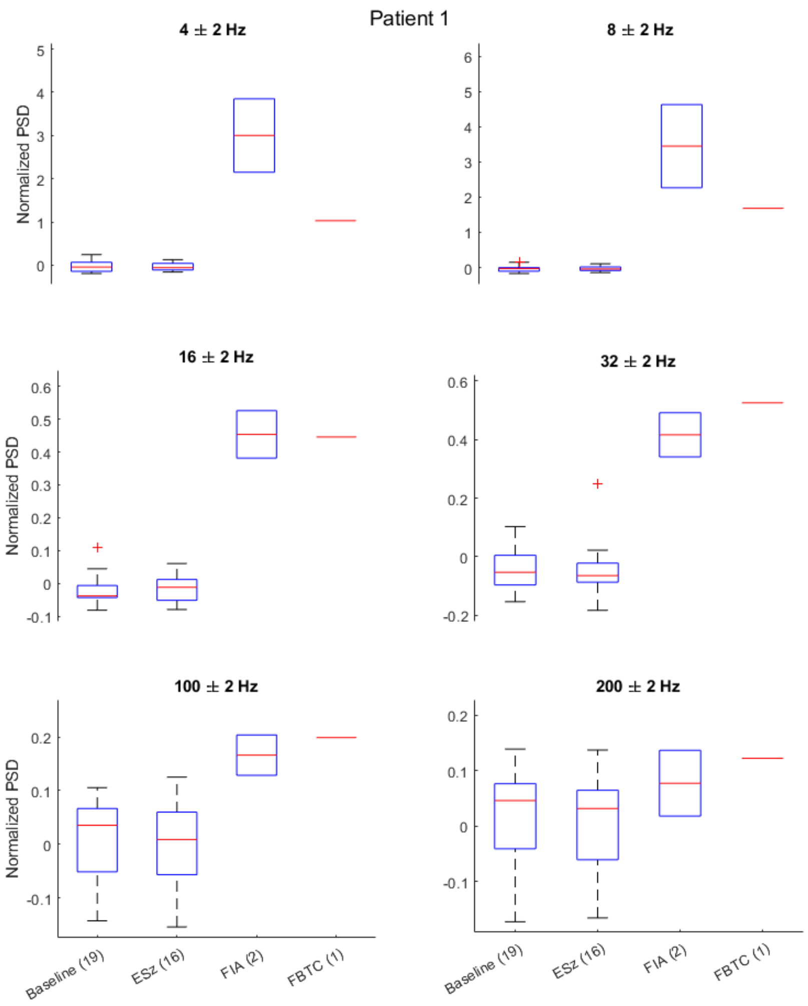

Figure S2: Results of Dunnett's test for patient 1 comparing the distribution of the average normalized power spectral density (PSD) during each seizure type (ESz, FIA, and FBTC) versus the matched baseline segment taken two minutes pre-ictal (PreSz). Results indicate no significant change in ANT-LFP. *P < 0.01, **P < 0.001.

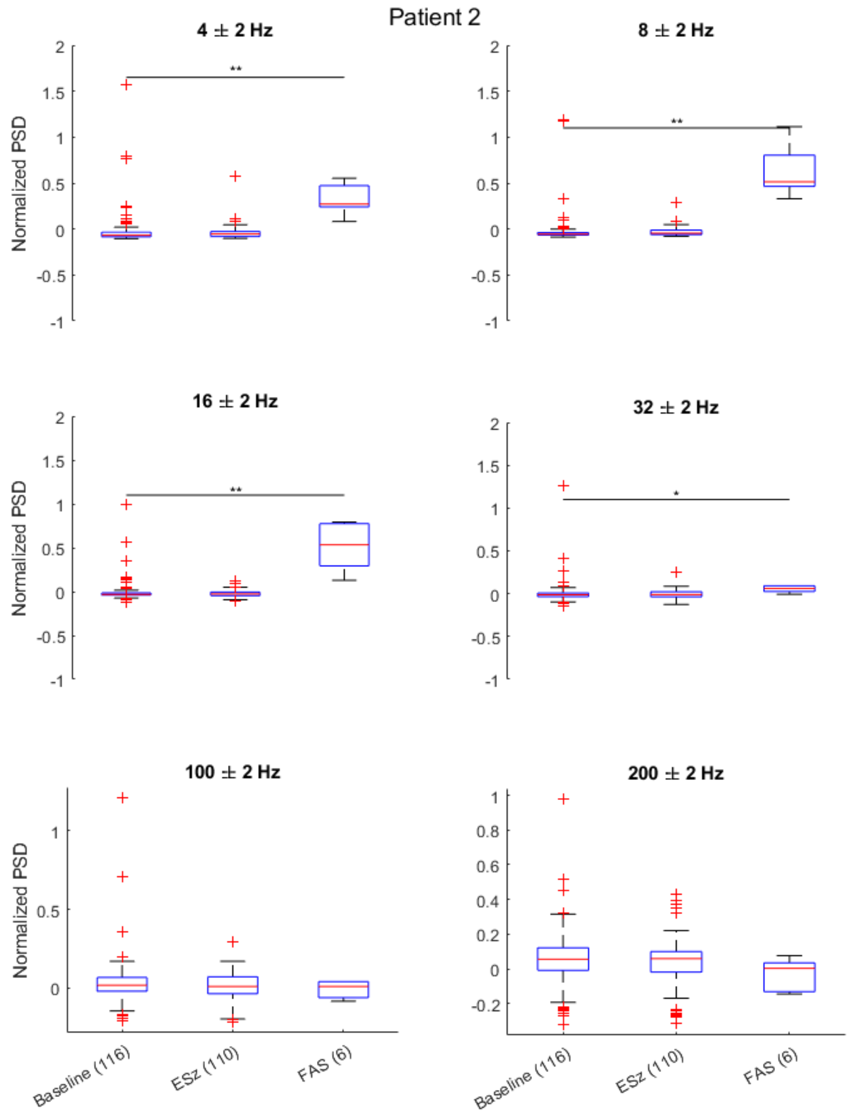

Figure S3: Results of Dunnett's test for patient 2 comparing the distribution of the average normalized power spectral density (PSD) during each seizure type (ESz and FAS) versus the matched baseline segment taken two minutes pre-ictal (PreSz). Results indicate an increase in ANT-LFP during FAS at 4, 8, 16, and 32 Hz. No change was observed in ESz. *P < 0.01, **P < 0.001.

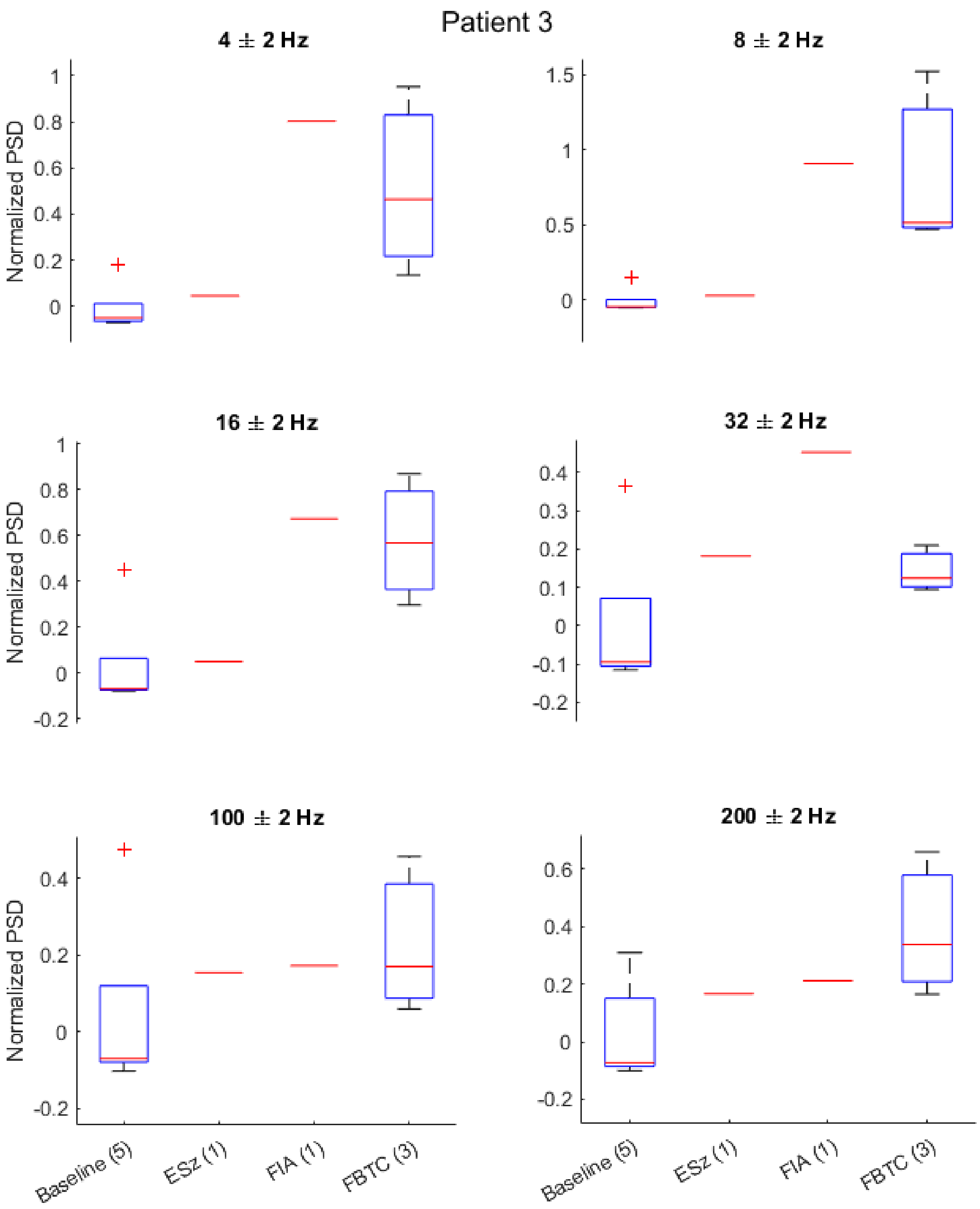

Figure S4: Results of Dunnett's test for patient 3 comparing the distribution of the average normalized power spectral density (PSD) during each seizure type (ESz, FIA, and FBTC) versus the matched baseline segment taken two minutes pre-ictal (PreSz). Results indicate no significant change in ANT-LFP. *P < 0.01, **P < 0.001.

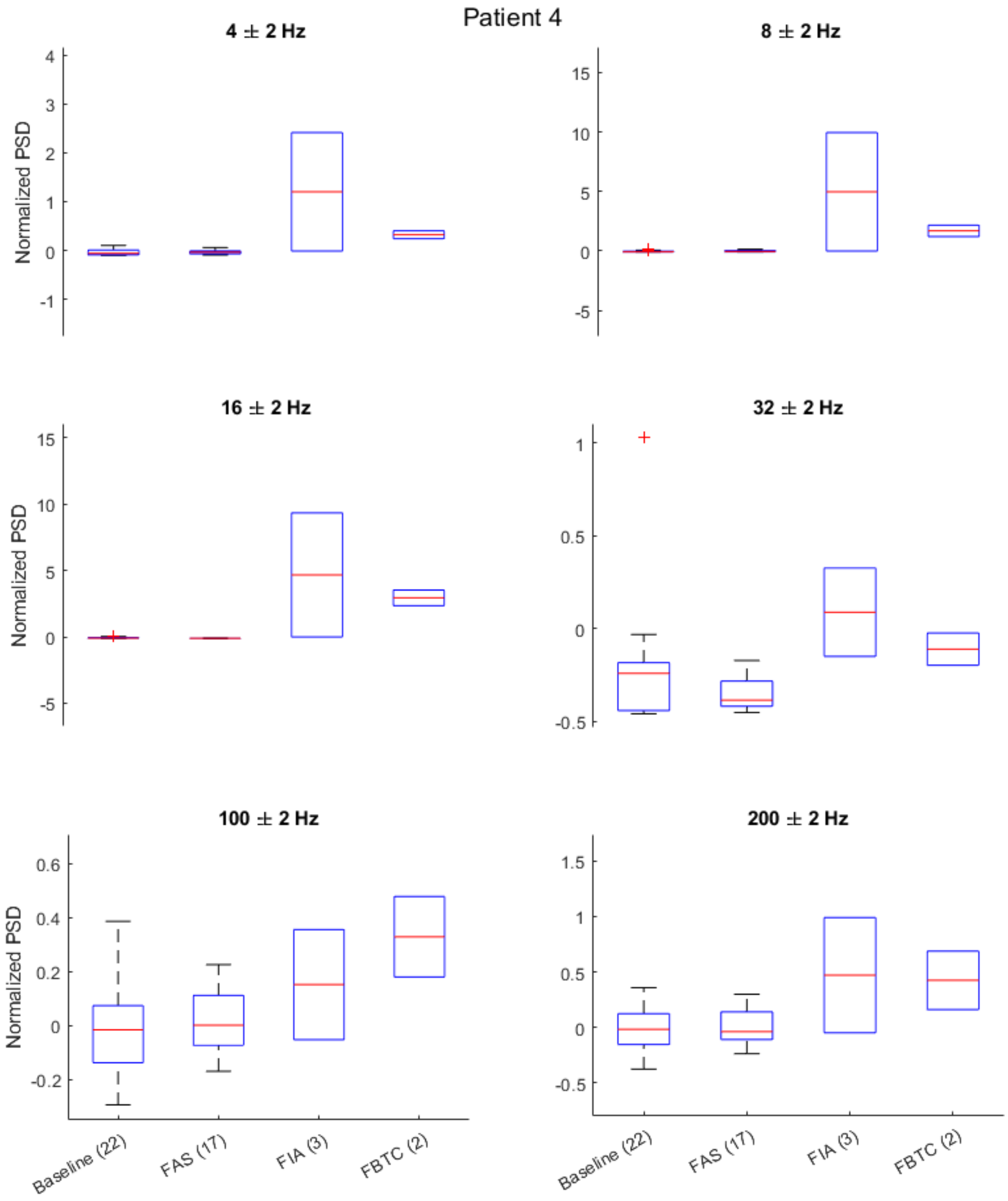

Figure S5: Results of Dunnett's test for patient 4 comparing the distribution of the average normalized power spectral density (PSD) during each seizure type (FAS, FIA, and FBTC) versus the matched baseline segment taken two minutes pre-ictal (PreSz). Results indicate no significant change in ANT-LFP. *P < 0.01, **P < 0.001.
